# *PAX3* is a novel tumor suppressor by regulating the activities of major signaling pathways and transcription factor FOXO3a in thyroid cancer

**DOI:** 10.18632/oncotarget.10753

**Published:** 2016-07-21

**Authors:** Wei Liu, Fang Sui, Jiazhe Liu, Meichen Wang, Sijia Tian, Meiju Ji, Bingyin Shi, Peng Hou

**Affiliations:** ^1^ Department of Endocrinology, The First Affiliated Hospital of Xi'an Jiaotong University, Xi'an, 710061, P.R. China; ^2^ Center for Translational Medicine, The First Affiliated Hospital of Xi'an Jiaotong University, Xi'an, 710061, P.R. China; ^3^ Key Laboratory for Tumor Precision Medicine of Shaanxi Province, The First Affiliated Hospital of Xi'an Jiaotong University, Xi'an, 710061, P.R. China

**Keywords:** thyroid cancer, PAX3, PI3K/Akt pathway, MAPK/Erk pathway, FOXO3a

## Abstract

Paired box 3 (*PAX3*) is expressed early during embryonic development in spatially restricted domains in the nervous system and in some mesodermally-derived structure. In recent years, it is found to be overexpressed in different types of cancer tissues and cell lines including glioblastomas, neuroblastomas, melanomas, rhabdomyosarcomas, Ewing sarcomas and gastric cancers, suggesting that it may function as an oncogene in these cancers. However, its role in thyroid cancer remains totally unclear. The aim of this study was to explore the functions and related molecular mechanism of PAX3 in thyroid tumorigenesis. Using quantitative RT-PCR (qRT-PCR) and Methylation-specific PCR (MSP) assays, we demonstrated that *PAX3* was frequently down-regulated by promoter methylation in both primary thyroid cancer tissues and thyroid cancer cell lines. In addition, our data showed that ectopic expression of PAX3 dramatically inhibited thyroid cancer cell proliferation, colony formation, migration and invasion, induced cell cycle arrest and apoptosis and retarded tumorigenic potential in nude mice. Mechanically, PAX3 exerted its tumor suppressor function by inhibiting the activity of major signaling pathways including the phosphatidylinositol-3-kinase (PI3K)/Akt and MAPK/Erk pathways, and enhancing expression and activity of transcription factor FOXO3a. Altogether, our findings provided insight into the role of PAX3 as a novel functional tumor suppressor in thyroid cancer through modulating the activities of PI3K/Akt and MAPK signaling pathways and transcription factor FOXO3a, and demonstrated that epigenetic alterations such as promoter methylation should be a major mechanism of PAX3 inactivation in this cancer.

## INTRODUCTION

The paired box (*PAX/Pax*) gene family is now recognized as potentially playing crucial roles in cellular proliferation, differentiation, migration and tissue development [1]. This family comprises nine members in humans (*PAX1*-*PAX9*) and mice (*Pax1*-*Pax9*), whereas they are further classified into four subgroups by structural similarity such as paired domain, homeodomain and octapeptide [2]. It is now clear that there is a potential link between aberrant expression of *PAX* genes in adult tissues and a wide variety of cancers by promoting or inhibiting tumorigenesis [2, 3]. As a member of *PAX* gene family, PAX3 has been found to be correlated with oncogenesis [4], and is upregulated and highly expressed in glioblastomas, neuroblastomas, melanomas, rhabdomyosarcomas, Ewing sarcomas and gastric cancers [5–10]. These observations suggest that *PAX3* may be a potential oncogene in tumorigenesis. However, the function of *PAX3* in thyroid cancer is unclear.

As the most common endocrine malignancy, thyroid cancer has been rapidly increasing in many regions of the world including China [11, 12]. Well-differentiated thyroid cancers (WDTCs), accounting for more than 90% of thyroid cancer, are composed of follicular thyroid cancer (FTC) and papillary thyroid cancer (PTC). Usually, the patients with WDTCs have an excellent prognosis and can be curved by surgical and radioiodinated therapy. However, approximately 10% of cases can develop into more aggressive and dedifferentiated forms of thyroid cancer, leading to recurrent disease and death [13]. Given that epigenetic silencing of tumor-associated genes by promoter hypermethylation exerts a fundamental role in tumorigenesis [14, 15], considerable efforts have been recently done to identify novel target genes, which are silenced by promoter methylation and function as a putative tumor suppressor in human cancers including thyroid cancer.

In this study, we demonstrated epigenetic silencing of *PAX3* by promoter methylation in a cohort of PTCs. A series of *in vitro* and *in vivo* studies showed that ectopic expression of PAX3 dramatically inhibited cell growth and invasiveness in thyroid cancer cells through repressing the activities of PI3K/Akt and MAPK/Erk pathways and promoting FOXO3a activity. These findings support that *PAX3* functions as an oncosuppressor in thyroid tumorigenesis.

## RESULTS

### Down-regulation of *PAX3* by promoter methylation in PTCs and thyroid cancer cell lines

**Figure 1:**
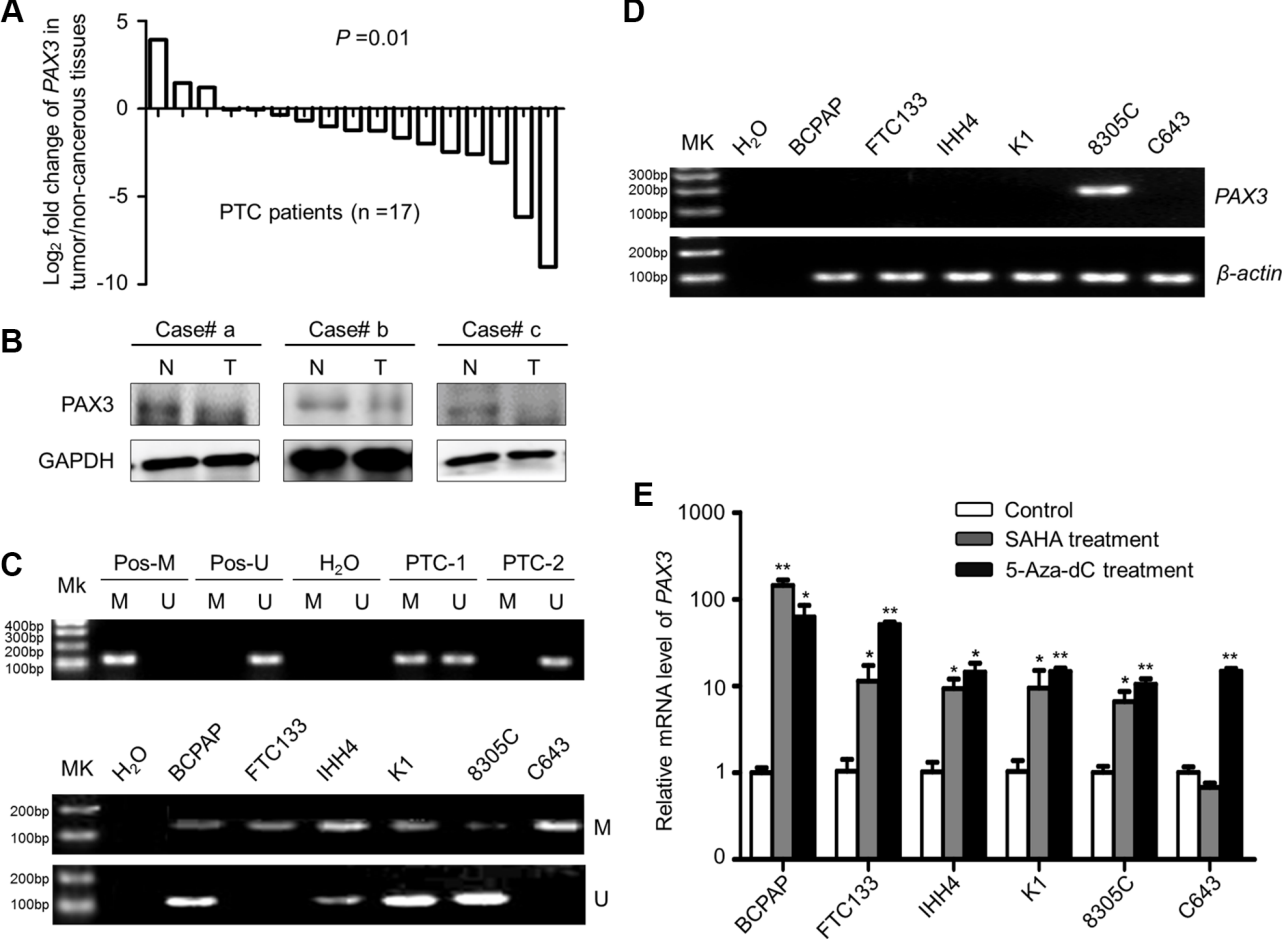
*PAX3* inactivation by promoter hypermethylation in primary PTCs and thyroid cancer cell lines (**A**) qRT-PCR assay was performed to evaluate mRNA expression of *PAX3* in primary PTCs and their matched non-cancerous thyroid tissues (*n* = 17). *PAX3* expression was normalized with *18S* rRNA levels. Data are shown as Log2 fold change of *PAX3* expression in tumor/non-cancerous tissues. (**B**) Western blotting was performed to analyze the protein levels of PAX3 in PTCs (T) and matched non-cancerous tissues (N). GAPDH was used as loading control. (**C**) Promoter methylation of *PAX3* in primary PTCs (upper panel) and thyroid cancer cell lines (lower panel) was determined with MSP assay. *In vitro* methylated DNA was used as positive control for methylated gene (Pos-M), bisulfite-modified normal leukocyte DNA as positive control for unmethylated gene (Pos-U), and H_2_O as blank control to confirm the specificity of MSP. Mk, DNA marker; M, methylated gene; U, unmethylated gene. PTC-1 and -2 present two PTC cases with different methylation status of *PAX3*. (**D**)*PAX3* expression in thyroid cancer cell lines was determined by conventional RT-PCR. *β-actin* was run as loading control. (**E**) *PAX3* expression was restored after treatment with 5-Aza-dC or SAHA. Its expression was determined by qRT-PCR. *18S* rRNA was used as a normalized control. Data were presented as mean ± SD. Statistically significant differences were indicated: *P < 0.05; **P < 0.01; ***P < 0.001.

We first examined mRNA expression of *PAX3* in 17 primary PTCs and matched non-cancerous thyroid tissues (control subjects) by using qRT-PCR approach. Although *PAX3* was not mainly expressed in thyroid epithelial cells, down-regulation of *PAX3* was still found in 14 of 17 (82.4%) PTCs as compared with control subjects (*P* = 0.01) (Figure 1A). Moreover, we also assessed the protein levels of PAX3 in PTCs and the matched non-cancerous tissues by western blotting. The results further demonstrated downregulation of PAX3 in PTCs compared with control subjects (Figure 1B). These data suggest epigenetic silencing of *PAX3* in thyroid cancer. Next, we attempted to evaluate promoter methylation of *PAX3* in a large cohort of PTCs by using MSP assay. Our data showed that *PAX3* methylation was found in 118/178 (66.3%) PTCs, whereas it was only found in 3/23 (13.0%) control subjects. Figure 1C (upper panel) exhibited methylation status of two representative PTC cases. Similar to the findings in primary PTCs, full or partial methylation was detected in all of six thyroid cancer cell lines (Figure 1C, lower panel). Accordingly, *PAX3* was silenced in five of six thyroid cancer cell lines except for 8305C (Figure 1D). To further clarify whether *PAX3* is epigenetically silenced in thyroid cancer cells, we treated these cell lines with 5-Aza-dC or SAHA, respectively. As shown in Figure 1E, 5-Aza-dC treatment significantly increased *PAX3* expression in all cell lines, further suggesting that transcriptional inactivation of *PAX3* was mediated by promoter methylation. Moreover, SAHA treatment also restored *PAX3* expression in most of cell lines except for C643. Taken together, these observations suggest that epigenetic silencing is one of the major causes underlying *PAX3* down-regulation in thyroid cancer.

### Inhibition of thyroid cancer cell growth by PAX3

**Figure 2:**
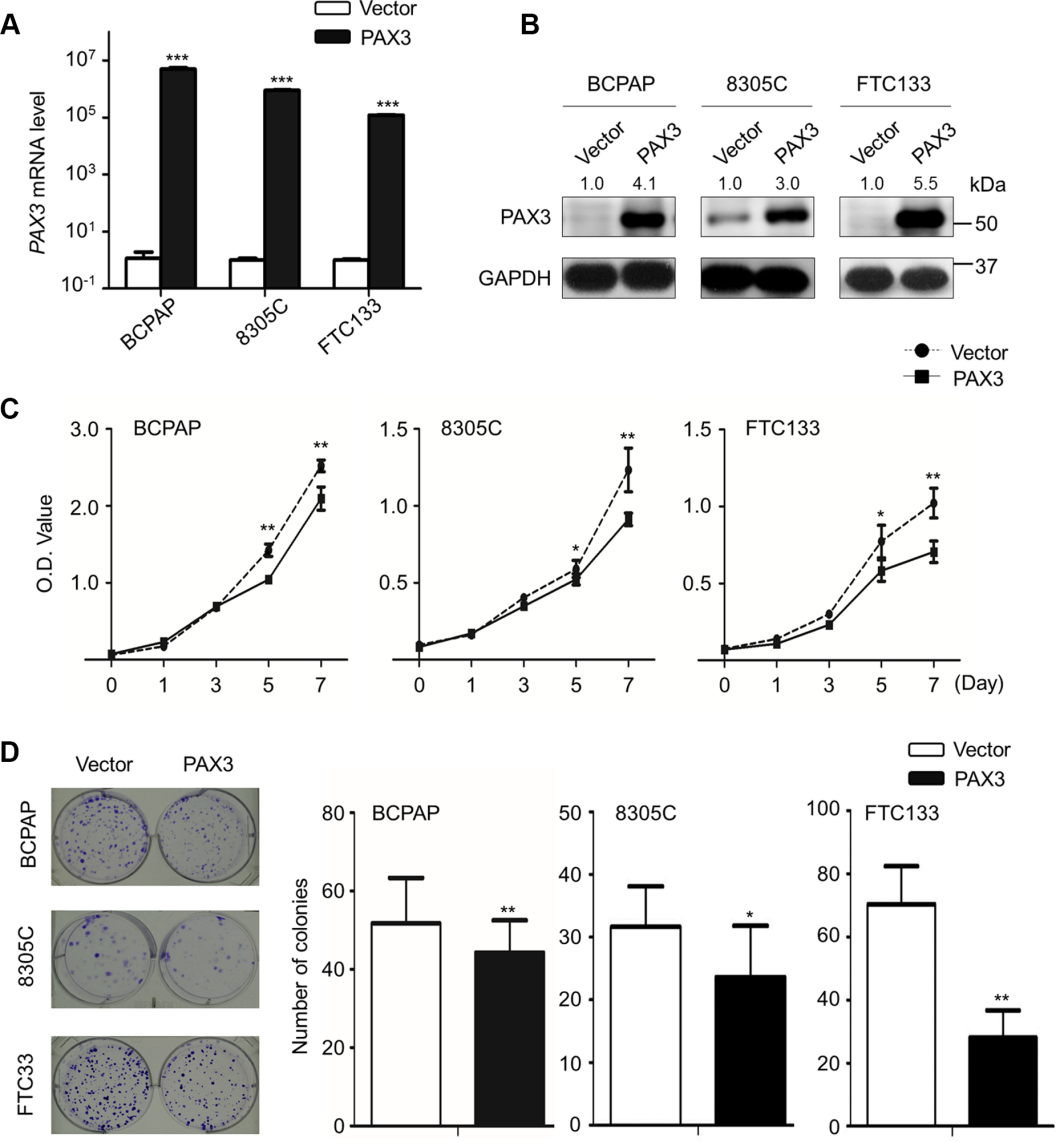
Inhibition of cell proliferation and colony formation by PAX3 in thyroid cancer cells Ectopic expression of *PAX3* mRNA (**A**) and protein (**B**) in thyroid cancer cell lines BCPAP, 8305C and FTC133 was evidenced by qRT-PCR and western blot assays, respectively. *18S* rRNA was used as a normalized control for qRT-PCR assay. GAPDH was used as loading control in western blot assay. (**C**) PAX3 re-expression significantly inhibited cell proliferation in thyroid cancer cells. (**D**) The effect of PAX3 re-expression on cell growth was further confirmed by colony formation assay. Left panel shows the representative images of colony formation in cells transfected with pcDNA3.1-PAX3 or empty vector. Quantitative analysis of colony numbers is shown in the right panel. Data were presented as mean ± SD. Statistically significant differences were indicated: *P < 0.05; **P < 0.01; ***P < 0.001.

Frequent inactivation of *PAX3* in thyroid cancer cells and PTC samples but not in normal tissues indicates that *PAX3* functions as an oncosuppressor in thyroid cancer. Next, we determined the effect of ectopic expression of PAX3 on cell growth in BCPAP, 8305C and FTC133 cells. PAX3 re-expression in these cells was verified by qRT-PCR (Figure 2A) and western blot (Figure 2B) assays. As shown in Figure 2C, PAX3 re- expression significantly inhibited cell proliferation as compared with controls. The growth-inhibitory effect of PAX3 was further confirmed by colony formation assay. The colonies number in PAX3*-*transfected group were decreased as compared with empty vector-transfected group in Figure 2D. Similar results were also found in the other three cell lines IHH4, K1 and C643 (Supplementary Figure S1). Collectively, our data suggest that PAX3 may exert tumor suppressor function.

### Induction of cell cycle arrest and apoptosis of thyroid cancer cells by PAX3

**Figure 3:**
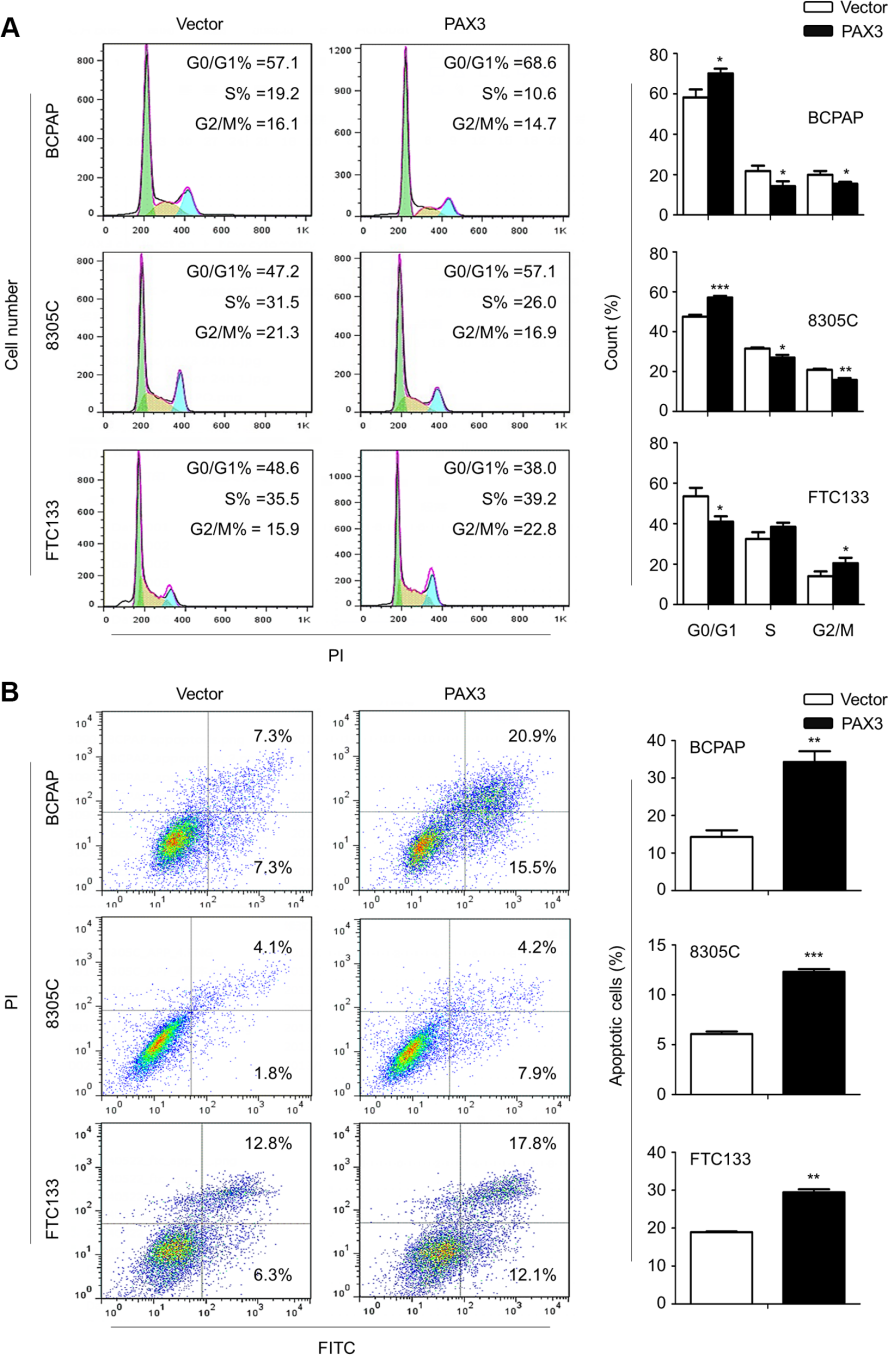
Induction of cell cycle arrest and apoptosis by PAX3 in thyroid cancer cells (**A**) BCPAP, 8305C and FTC133 cells were transiently transfected with pcDNA3.1-PAX3 or empty vector, respectively. DNA content was measured by flow cytometry to determine cell cycle fractions. The fraction of cells in each cell cycle phase was indicated in the figures. (**B**) Apoptotic cells including early and late apoptotic cells were measured 48 h after transfection by flow cytometry analysis of Annexin V-FITC/PI double-labelled cells. The data were presented as mean ± SD of values from three independent experiments. Statistically significant differences were indicated: *P < 0.05; **P < 0.01; ***P < 0.001.

We next tested the effects of PAX3 re-expression on cell cycle arrest and apoptosis. As shown in Figure 3A, cell cycle was blocked at G0/G1 phase in BCPAP and 8305C cells, and at G2/M phase in FTC133 cells when these cells were transfected with PAX3 expression plasmid (pcDNA3.1-PAX3) as compared with empty vector. In addition, ectopic expression of PAX3 dramatically induced cell apoptosis compared with controls (Figure 3B).

### Inhibition of tumor growth by PAX3 *in vivo*

**Figure 4:**
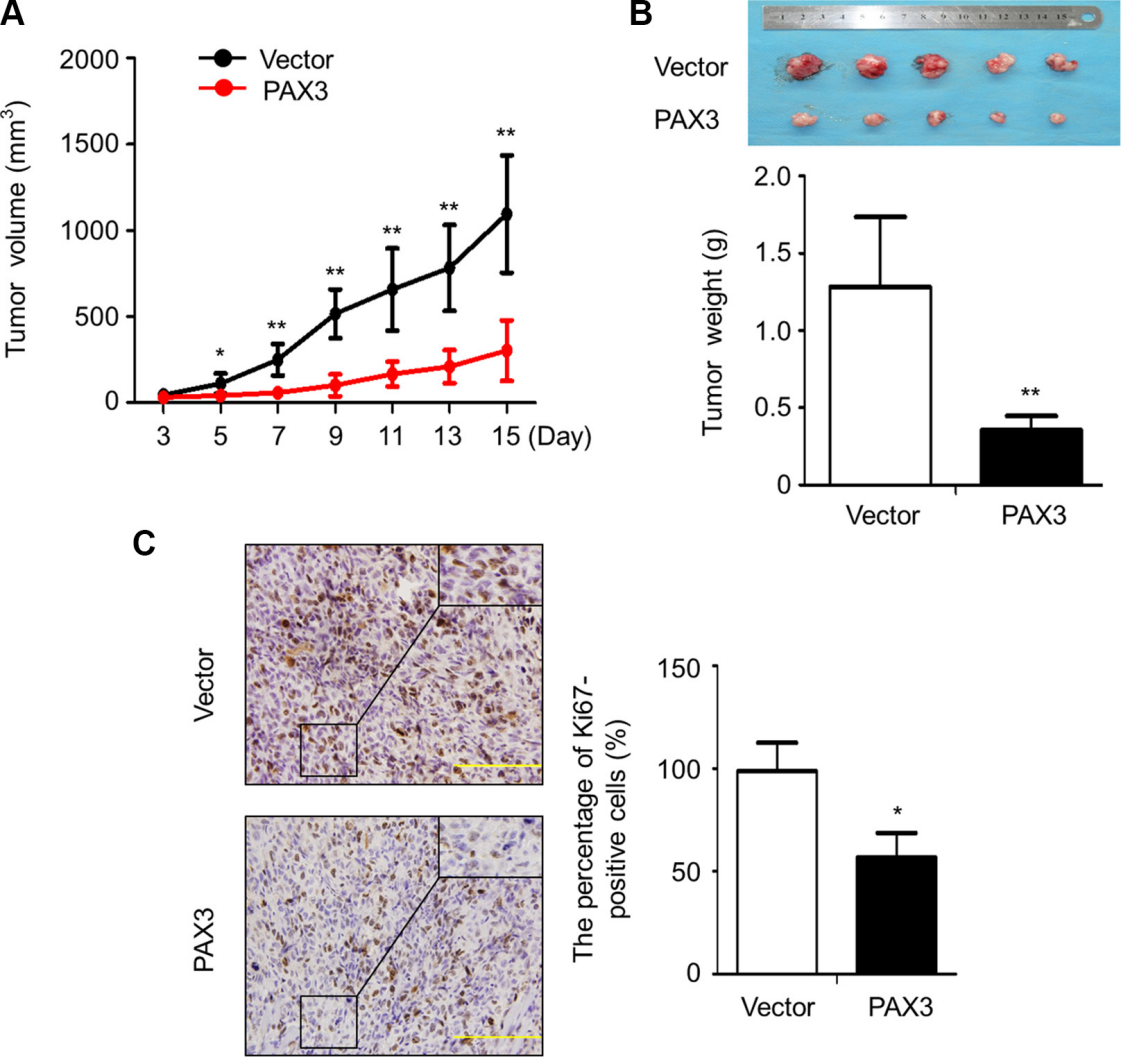
Inhibition of the xenograft tumor growth by PAX3 (**A**) Subcutaneous tumor growth curve of PAX3-transfected FTC133 cells in nude mice was compared with empty vector-transfected cells. The PAX3-FTC133 group showed a retarded tumor growth compared to control group. Data are shown as mean ± SD (*n* = 5/group). (**B**) A representative picture for tumor growth of PAX3- or empty vector-transfected cells in nude mice (upper panel). Histogram represents mean of tumor weight from the PAX3-FTC133 and control groups (lower panel). Data are shown as mean ± SD (*n* = 5/group). (**C**) Shown is representative Ki-67 staining of xenograft tumors from PAX3-FTC133 and control groups (left panels). Histogram represents mean ± SD of the percentage of Ki-67-positive cells from 5 microscopic fields in each group (right panel). Statistically significant differences were indicated: *P < 0.05; **P < 0.01.

Given *in vitro* growth-inhibitory activity of PAX3, we next assessed the impact of PAX3 re-expression on tumor growth in nude mice. As shown in Figure 4A, tumor induced by PAX3*-*transfected FTC133 cells showed significantly longer latency and smaller mean tumor volume than tumors induced by control cells. After 15 days post-transplantation, the xenograft tumors were peeled off and weighted. The average weight of the tumors in the PAX3-FTC133 group was significantly less compared with control group (*P* = 0.009; Figure 4B). To assess the proliferation index in the xenograft tumor, tumor sections were stained with anti-Ki-67 antibody. The percentage of Ki-67 positive cells was dramatically decreased in the PAX3-FTC133 group compared with control group (*P* = 0.02; Figure 4C). These results further support that PAX3 is a potential oncosuppressor in thyroid cancer.

### Inhibition of thyroid cancer cell migration and invasion by PAX3

**Figure 5:**
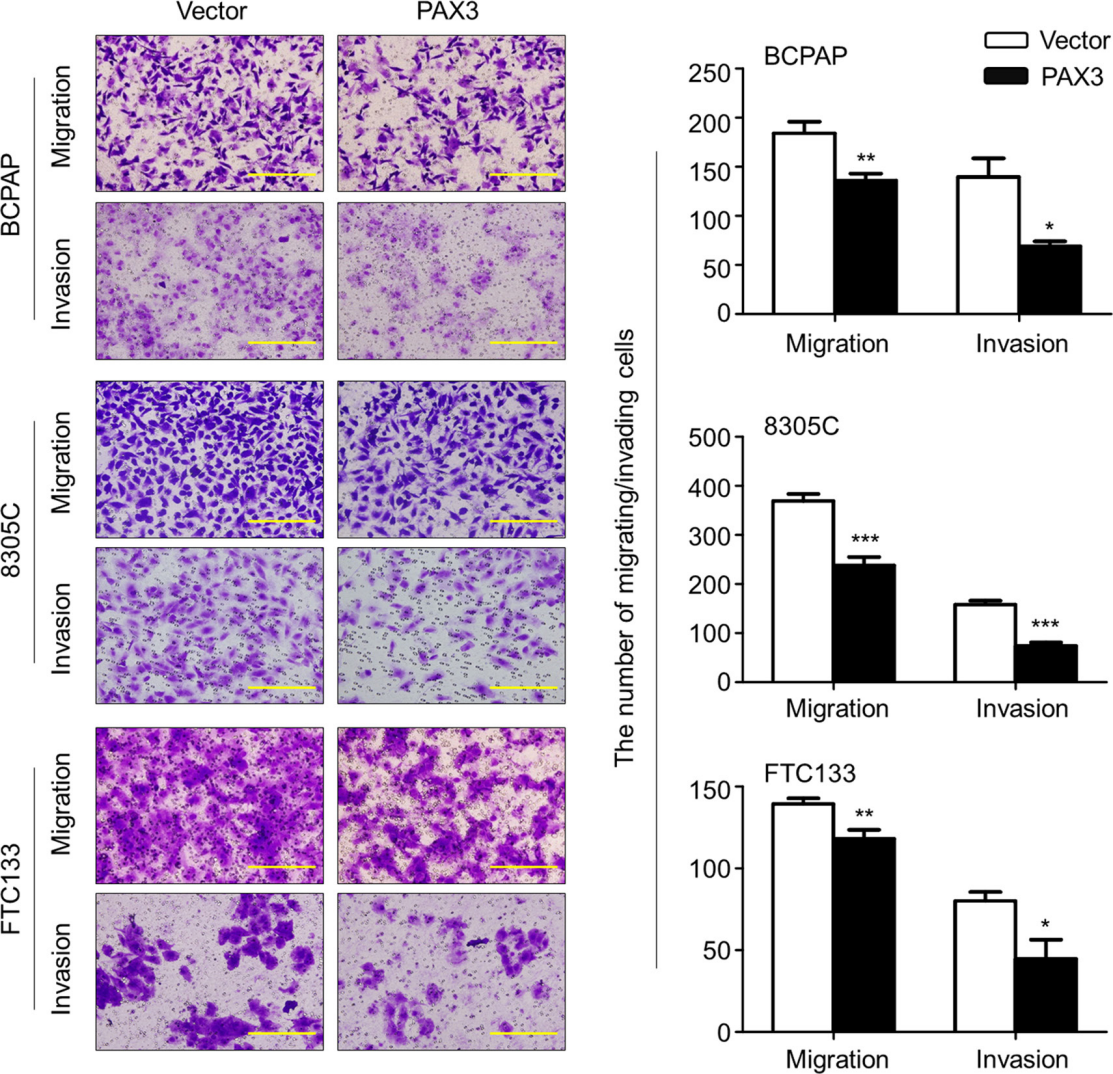
Inhibition of thyroid cancer cell migration and invasion by PAX3 Ectopic expression of PAX3 inhibited cell migration and invasion in BCPAP, 8305C and FTC133 cells. The representative images of migrated/invaded cells (left panels). Histograms, corresponding to left panels, show means ± SD of cell numbers from three independent assays (right panels). Statistically significant differences were indicated: *P < 0.05; **P < 0.01; ***P < 0.001.

Given that metastasis is the major cause of death in thyroid cancer, we next evaluated the impact of PAX3 re-expression on thyroid cancer cell migration and invasion. As shown in Figure 5 and Supplementary Figure S2, the number of migrated cells in the PAX3-transfected cells was significantly decreased than that in the control cells. Moreover, the invasion assay showed that the number of PAX3-transfected cells passing through the Matrigel-coated membrane was significantly decreased as compared with control cells (Figure 5). These data suggest that there is a potential link between epigenetic inactivation of *PAX3* and metastatic phenotypes in thyroid cancer.

### PAX3 regulates the activities of major pathways and FOXO3a in thyroid cancer

**Figure 6:**
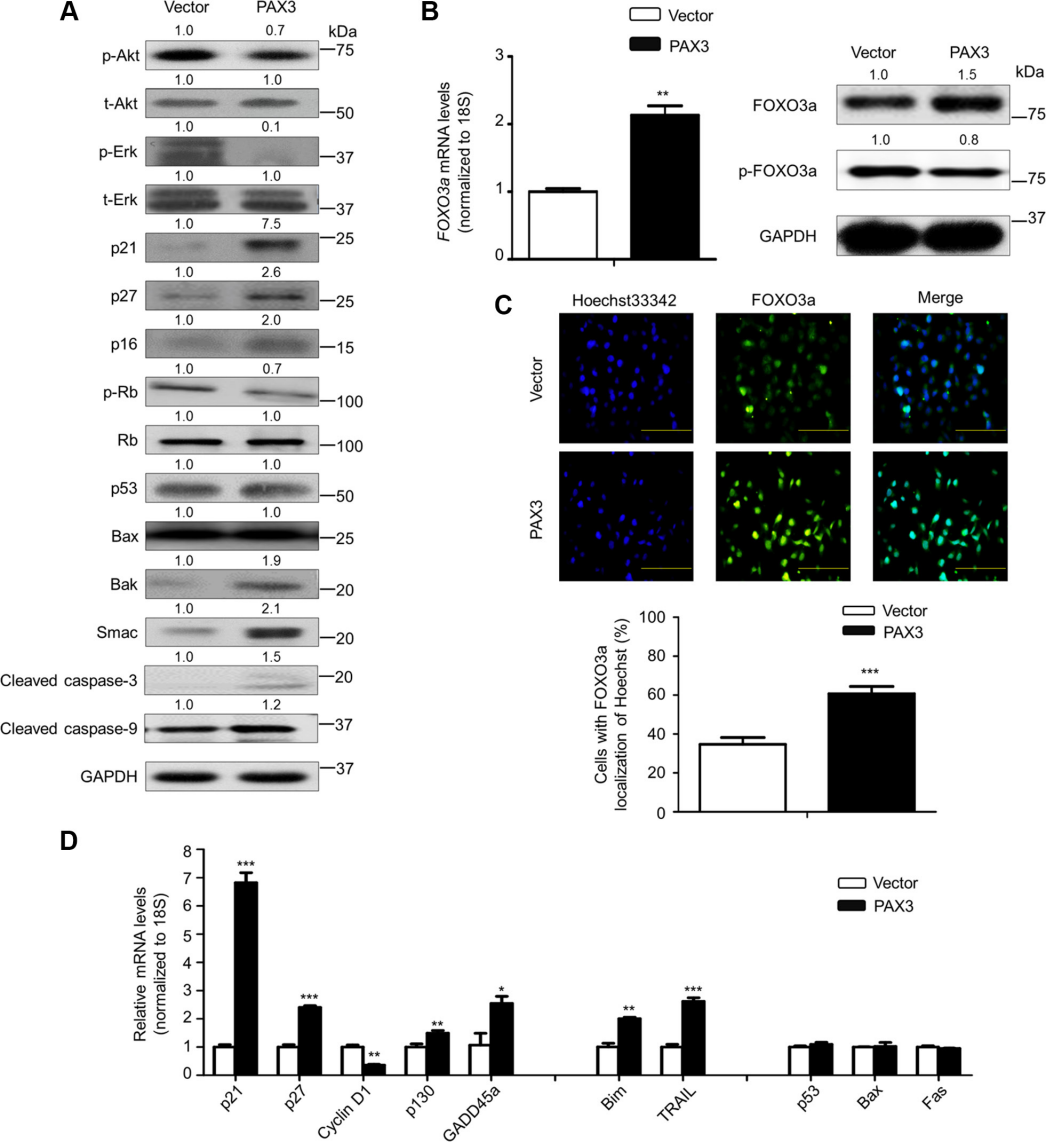
Modulation of the activities of major signaling pathways and FOXO3a by PAX3 in thyroid cancer cells (**A**) The indicated cells were lysed and lysates were subjected to western blot analysis. The antibodies against phospho-Akt (p-Akt), total Akt (t-Akt), phospho-Erk (p-Erk) and total Erk (t-Erk) were used to determine the effect of PAX3 re-expression on the activities of PI3K/Akt and MAPK/Erk pathways. The antibodies against p21, p27, p16, phospho-Rb (p-Rb) and Rb were used to determine the effect of PAX3 re-expression on the regulation of cell cycle. The antibodies against p53, Bax, Bak, Smac, activated caspase-3 and -9 were used to determine the effect of PAX3 re-expression on cell apoptosis. GAPDH was used as loading control. (**B**) Total RNA and protein were extracted from the indicated cells. qRT-PCR assay was performed to determine the effect of PAX3 re-expression on *FOXO3a* expression (left panel). Western blotting was used to assess the effect of PAX3 re-expression on the levels of FOXO3a protein and phosphorylated FOXO3a (p-FOXO3a) (right panel).*18S* rRNA was used as an endogenous control for qRT-PCR assay. GAPDH was used as loading control for western blot analysis. (**C**) Immunofluorescence assay was used to test the effect of PAX3 re-expression on the expression and cellular localization of FOXO3a (upper panel). Colocalization of FOXO3a with Hoechst33342 was quantified in low panel. Green color represents FOXO3a fluorescence and blue color represents Hoechst33342 staining for nuclei. (**D**) qRT-PCR assay was used to test the effect of PAX3 re-expression on the expression of a set of cell cycle- and apoptosis-related genes. Expression levels of these genes were normalized with *18S* rRNA levels. Data were presented as mean ± SD. Statistically significant differences were indicated: *P < 0.05; **P < 0.01; ***P < 0.001.

Given the importance of PI3K/Akt and MAPK/Erk pathways in thyroid tumorigenesis [14], we thus tested the effect of ectopic expression of PAX3 on the activities of these two pathways. As shown in Figure 6A, PAX3 re-expression remarkably decreased the levels of phosphorylation of Akt and Erk in BCPAP cells. Similar results were also found in the other two cell lines 8305C and FTC133 (Supplementary Figure S3). These data suggest that PAX3 functions as a tumor suppressor by inhibiting the activities of PI3K/Akt and MAPK/Erk pathways.

To explore the mechanism underlying PAX3-mediated cell cycle arrest, we evaluated the impact of PAX3 re-expression on the expression of cell cycle regulatory factors such as p21, p27, p16 and phosphorylated Rb (p-Rb). As expected, PAX3 re-expression significantly increased the expression of p21, p27 and p16 compared with the control. Moreover, we also found that ectopic expression of PAX3 remarkably up-regulated the mRNA levels of *p16*, but not *Rb* in BCPAP cells (Supplementary Figure S4). A previous study has demonstrated that inhibition of MAPK/Erk pathway can up-regulate *p16* expression through promoter demethylation via down-regulating DNMT1 expression in colon cancer cells [16]. This was supported by our data that inhibition of MAPK/Erk pathway using MEK inhibitor GSK1120212 cascade up-regulated *p16* expression, down-regulated the expression of *DNMT1* and *DNMT3b*, and the expression of PRC2 components EZH2 and SUZ12 contributing to decreased levels of H3K27me3 (Supplementary Figure S5). Altogether, we speculated that PAX3 regulated *p16* expression through epigenetic mechanisms such as promoter methylation and H3K27me3 via inhibition of MAPK/Erk cascade. According to increased expression of p16 upon PAX3 re-expression, the expression of p-Rb protein was dramatically reduced in PAX3-tranfected cells compared with control cells (Figure 6A), suggesting the role of the p16/Rb signaling in PAX3-mediated cell cycle arrest. Next, we investigated the impact of PAX3 re-expression on the levels of apoptosis-associated proteins including p53, Bax, Bak, Smac, activated caspase-3 and -9. The results showed that the levels of p53 and Bax were not changed by ectopic expression of PAX3, whereas the levels of Bak, Smac, activated caspase-3 and -9 were significantly increased in PAX3-transfected cells compared with control cells (Figure 6A).

Given that p21, a major downstream target of p53, can be up-regulated by PAX3 through a p53-independent pathway, we thus suppose that PAX3 may modulate p21 expression through unknown mechanisms. It has been reported that transcription factor FOXO3a, another downstream effector of the PI3K/Akt and MAPK/Erk pathways [17], can also regulate a panel of downstream genes at transcriptional levels. Some of them are related with cell cycle arrest and apoptosis [18, 19]. Thus, we next evaluated the influence of PAX3 re-expression on FOXO3a activity. As shown in Figure 6B, ectopic expression of PAX3 up-regulated FOXO3a expression at both transcriptional and post-transcriptional levels. Moreover, our data showed that PAX3 restoration slightly suppressed phosphorylation of FOXO3a in BCPAP cells (Figure 6B). Similar results were also found in another thyroid cancer cell line FTC133 (Supplementary Figure S6). There is strong evidence showing that FOXO3a is post-transcriptionally regulated by some signaling pathways such as PI3K/Akt pathway [17, 20, 21]. Activation of Akt phosphorylates FOXO3a at several conservative sites such as Ser 253, Thr 32 and Ser 315 and initiates its nucleocytoplasmic shuttling and ultimately targets it for proteasome degradation [21]. In addition, our results showed that PAX3 re-expression also caused a significant nuclear accumulation of non-phosphorylated FOXO3a in BCPAP cells (Figure 6C). FOXO3a may serve as a transcription factor to regulate the expression of its targets when it is localized in the nucleus [22, 23]. Thus, we next tested the effect of ectopic expression of PAX3on expression of a set of FOXO3a downstream targets, including cell cycle-related genes such as *p21*, *p27*, *cyclin D1*, *p130* and *GADD45a*, and apoptosis-related genes such as *Bim* and *TRAIL*. As shown in Figure 6D, PAX3 re-expression significantly up-regulated the expression of *p21*, *p27*, *p130*, *Bim* and *TRAIL*, and down-regulated *cyclin D1* expression in BCPAP cells. Moreover, we also evaluated the effect of ectopic expression of PAX3 on expression of *p53* and its two downstream target genes *Bax* and *Fas* in the same cell line. As expected, PAX3 re-expression did not affect the expression of these two genes (Figure 6D). Notably, we accidently found that PAX3 re-expression up-regulated the expression of transcription factor ZIC1 in BCPAP cells (Supplementary Figure S7). Our previous study has demonstrated that ZIC1 contributes to cell cycle arrest and apoptosis through a p53-independent pathway via enhancing FOXO3a transcriptional activity [24]. Thus, we speculated that PAX3 also regulated the expression and activity of FOXO3a possibly by promoting ZIC1 expression via other unknown mechanisms.

Given that oncogenic function of PAX3 has been previously reported in other cancers such as glioblastomas and melanomas [5, 7], we attempted to determine the role of PAX3 in glioblastoma cell line U251 and melanoma cell line A375. As shown in Supplementary Figure S8, our data showed that PAX3 re-expression increased phosphorylation of Akt and FOXO3a in U251 cells and phosphorylation of Erk in A375 cells, suggesting that PAX3 plays a tumor-promoting role in glioblastomas and melanomas probably through activating these signaling pathways. These results further demonstrate that PAX3 indeed plays the distinct biological roles in different types of cancer.

### Inhibition of the process of epithelial-mesenchymal transition (EMT) and the expression of metastasis-associated genes by PAX3

**Figure 7:**
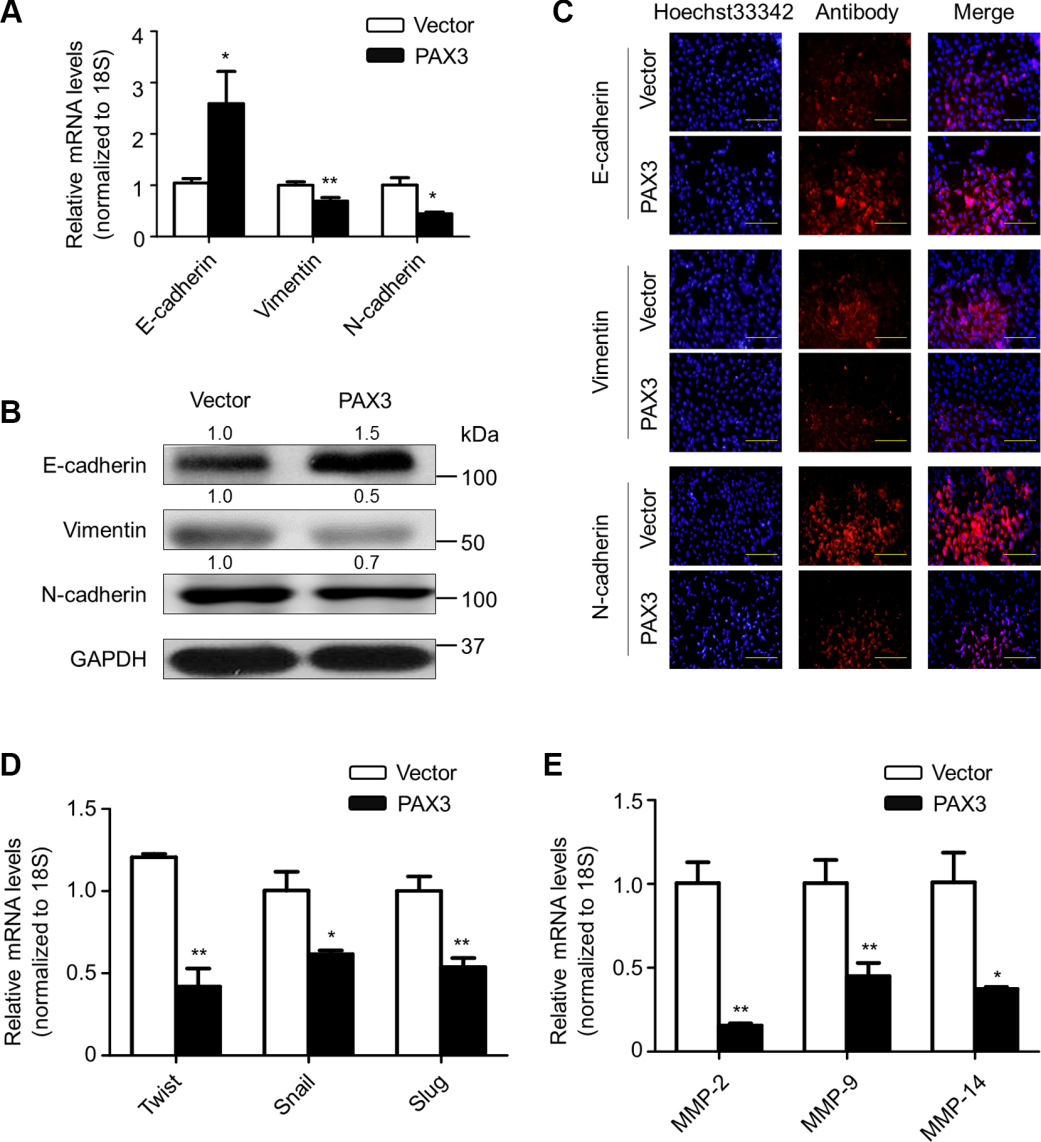
Inhibition of EMT process and the expression of metastasis-associated genes by PAX3 in thyroid cancer cells To test the effect of PAX3 re-expression on the process of EMT, the expression of E-cadherin, Vimentin and N-cadherin was determined at both mRNA (**A**) and protein (**B**) levels in the indicated cells by using qRT-PCR and western blot assays, respectively. *18S* rRNA was used as an endogenous control for qRT-PCR assay. GAPDH was used as loading control. (**C**) Immunofluorescence staining was performed to assess the expression of E-cadherin, Vimentin and N-cadherin proteins in the indicated cells. Red color represents target protein fluorescence and blue color represents Hoechst33342 staining for nuclei. (**D**) qRT-PCR assay was used to test the effect of PAX3 re-expression on mRNA expression of E-cadherin transcription suppressors *Twist*, *Snail* and *Slug* in the indicated cells. (**E**) qRT-PCR assay was also used to evaluate the effect of PAX3 re-expression on expression of metastasis-related genes *MMP*-*2*, -*9* and -*14* in the indicated cells. *18S* rRNA was used as a normalized control. Data were presented as mean ± SD. Statistically significant differences were indicated: *P < 0.05; **P < 0.01.

Given that EMT is a biological process inducing migratory and invasive capabilities and promoting tumor metastasis [25], we thus attempted to clarify the correlation between PAX3 and the EMT process in this study. As shown in Figure 7A and 7B, PAX3 re-expression substantially increased E-cadherin expression, whereas decreased the expression of Vimentin and N-cadherin at transcriptional and translational levels, as supported by immunofluorescence assay in Figure 7C. Next, we tested the effect of ectopic expression of PAX3 on the expression of E-cadherin transcription suppressor including Twist, Snail and Slug. As shown in Figure 7D, PAX3 re- expression dramatically inhibited expression of *Twist*, *Snail* and *Slug* in BCPAP cells.

Given the importance of matrix metalloproteinases (MMPs) in tumor metastasis [26], we also tested the ectopic expression of PAX3 on the expression of *MMP*-*2*, –*9* and –*14* in BCPAP, 8305C and FTC133 cells. As expected, PAX3 re-expression dramatically inhibited their expression (Figure 7E). Similar results were also observed in the other two cell lines 8305C and FTC133 (Supplementary Figure S9). Collectively, our findings unravel that PAX3 inhibits thyroid cancer cell metastasis through suppressing EMT process and the expression of metastasis-associated genes.

## DISCUSSION

In this study, we found that *PAX3* was frequently down-regulated in PTC samples compared with control subjects, suggesting that *PAX3* may be a potential oncosuppressor in thyroid cancer. Further studies revealed that *PAX3* inactivation was strongly related with promoter methylation. This was supported by our data that demethylation treatment significantly up-regulated *PAX3* expression in thyroid cancer cells. In addition, we found that SAHA treatment restored *PAX3* expression in these cells, indicating that histone deacetylation may also contribute to *PAX3* inactivation in addition to promoter methylation in thyroid cancer cells.

*PAX3* inactivation may abrogate tumor suppression, and promote thyroid tumorgenesis. Thus, we tested the role of PAX3 in thyroid cancer cells by a series of *in vitro* and *in vivo* experiments. Our data first demonstrated strong tumor suppressive activity of PAX3 in thyroid cancer through inhibiting cell growth and invasiveness. To better understand the mechanism of PAX3 as an oncosuppressor in thyroid cancer, we tested the effect of PAX3 re-expression on the PI3K/Akt and MAPK cascades in thyroid cancer cells. These two pathways as major therapeutic targets play an important role in thyroid tumorigenesis [14]. We found that ectopic expression of PAX3 strongly reduced phosphorylation of Akt and Erk, supporting that PAX3 exerts tumor suppressor function in thyroid cancer through modulating the activity of the PI3K/Akt and MAPK/Erk pathways.

It is clear that p53 is an important determinant factor of cell cycle and apoptosis in tumorigenesis. In this study, we tested the effect of PAX3 on p53 signaling. Our data showed that PAX3 re-expression did not change the expression of p53 and its targets *Bax* and *Fas*, whereas remarkably affected the expression of a set of cell cycle and apoptosis-associated genes. For example, p21, a well-known downstream target of p53, was increased by PAX3 at both mRNA and protein levels. Thus, we speculate that PAX3 induces cell cycle arrest and apoptosis through a p53-independent mechanism. Indeed, in this study, we found that PAX3 re-expression significantly increased the expression and activity of transcription factor FOXO3a. Accumulated evidences have demonstrated that FOXO3a plays an important role as a critical transcription factor in tumorigenesis through transcriptionally regulating cell cycle- and apoptosis-related genes in a p53-independent manner [18, 19, 22, 24]. This was supported by our findings that ectopic expression of PAX3 markedly up- regulated FOXO3a downstream target genes, such as *p21*, *p27*, *p130*, *Bim* and *TRAIL*, and down-regulated another target gene *cyclin D1* in thyroid cancer cells. These genes have been well demonstrated to be involved in regulation of cell cycle progression and apoptosis during tumorigenesis [18, 19]. Taken together, given that tumor suppressor p53 is frequently inactivated by missense mutations or deletion in undifferentiated thyroid cancers [27, 28], restoring or increasing FOXO3a transcriptional activity should be an effective strategy for the treatment of such cancers. In addition, our data showed that PAX3 up-regulated p16 expression, subsequently resulting in inhibition of Rb phosphorylation. These results suggest that PAX3 also induces cell cycle arrest in thyroid cancer cells by modulating p16/Rb/E2F signaling.

FOXO3a can also be post-transcriptionally regulated by the PI3K/Akt or MAPK/Erk pathways [17]. The PI3K/Akt signal-mediated phosphorylation of FOXO3a can not only decrease its transcriptional activity through disrupting its interaction with gene promoter, but also promote its proteasome degradation [17, 22]. There are evidences showing that FOXO3a is inactivated by over-activation of PI3K/Akt cascade [14, 19, 22]. In addition, aberrant MAPK/Erk signaling can also phosphorylate FOXO3a at multiple residues, subsequently promoting its degradation by the proteasome pathway [20]. Given that phosphorylation of Akt and Erk was strongly inhibited by PAX3, we conclude that PAX3 enhances FOXO3a transcriptional activity by suppression of the PI3K/Akt and MAPK/Erk cascades. Strikingly, we found that ectopic expression of PAX3 up-regulated the expression of ZIC1, a zinc-finger transcription factor, in this study. We have previously demonstrated that ZIC1 not only inhibits phosphorylation of Akt and Erk, but also increases FOXO3a transcriptional activity in thyroid cancer cells [24]. However, the mechanism underlying the regulation of ZIC1 by PAX3 remains to be explored. Collectively, these results suggest that PAX3 regulates the expression and activity of FOXO3a through multiple mechanisms in thyroid cancer.

The EMT, as a vital step of tumor transformation cascade, is closely associated with cancer progression and metastasis [25]. In this study, we found that PAX3 re-expression up-regulated epithelial marker E-cadherin accompanying with decreased expression of E-cadherin transcription suppressors *Snail*, *Slug* and *Twist*. Additionally, it down-regulated mesenchymal markers Vimentin and N-cadherin. These findings reveal that PAX3 inhibits cell metastasis by repressing EMT process in thyroid cancer. FOXO3a has been demonstrated to rescue E-cadherin from Snail and Twist mediated inhibition, resulting in the suppression of EMT [29, 30]. It is also clear that multiple complex signaling systems such as PI3K/Akt and MAPK/Erk pathways are required for induction of EMT [31, 32]. Thus, we can conclude that PAX3 inhibits the process of EMT by blocking the PI3K/Akt and MAPK/Erk cascades and enhancing expression and activity of FOXO3a in thyroid cancer cells. Moreover, we demonstrated that ectopic expression of PAX3 in thyroid cancer cells strongly inhibited the expression of *MMP*-*2*, -*9* and -*14*, suggesting that PAX3-induced metastasis suppression is also related with the activity of MMPs.

The previous studies have reported that PAX3 may play an oncogenic function in some cancers such as glioblastomas and melanomas [5, 7], whereas our data suggest that PAX3 acts as an oncosuppressor in thyroid cancer. Thus, we speculate that PAX3 derived from different types of cells or tissues may play distinct functions in tumorigenesis. In general, the binding site of transcription factor PAX3 is conservative in different tissues or cells. However, its specific function may be dependent on other factors. For example, PAX3 may form a complex with other nuclear factors and such interaction enhance or repress its transcriptional activity and defines the specificity of its targets. In addition, certain posttranslational modifications such as ubiquitinylation, phosphorylation or acetylation may also affect the activity of PAX3 by regulating its cellular localization, DNA-binding activity and stability.

**Figure 8:**
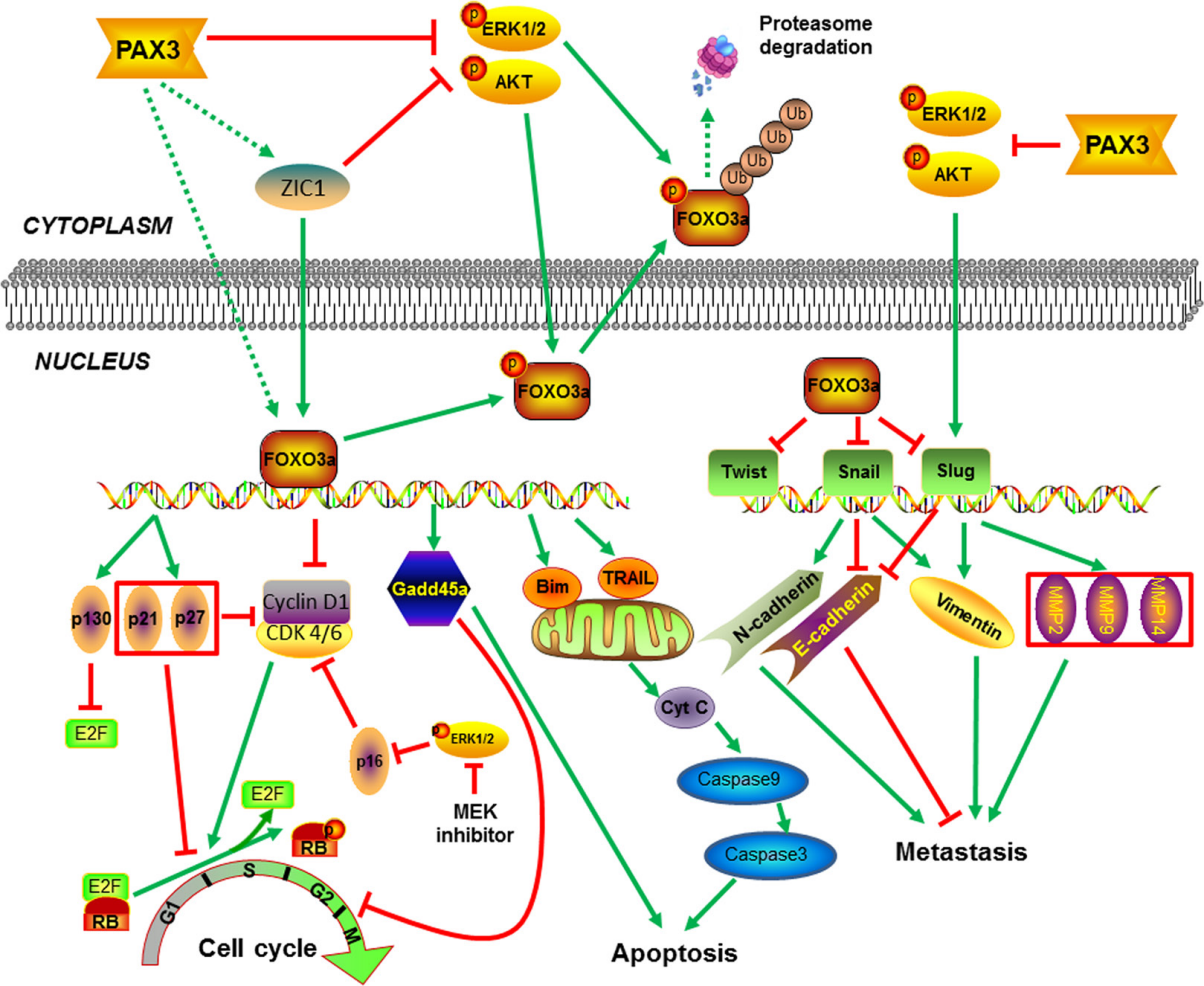
Schematic model of molecular mechanisms underlying tumor suppressive activity of PAX3 in thyroid cancer PAX3 increases the expression of *FOXO3a* at transcriptional levels through up-regulating ZIC1 expression or other unknown mechanisms. Meanwhile, PAX3 inhibits phosphorylation of FOXO3a, nuclear-cytoplasmic translocation and proteasome degradation through blocking the activity of PI3K/Akt and MAPK pathways, subsequently enhancing FOXO3a transcriptional activity in thyroid cancer cells. Downstream targets of FOXO3a exert tumor suppressive function through induction of cell cycle arrest and apoptosis, and inhibition of tumor metastasis. Moreover, PAX3 can also regulate cell cycle through modulating p16/Rb/E2F pathway via inhibition of major signaling pathways such as MAPK/Erk cascade.

In summary, we demonstrate that PAX3 is a novel oncosuppressor, and is frequently inactivated by promoter methylation in thyroid cancer. Our results are consistent with a model (Figure 8), in which PAX3 inhibits thyroid tumorigenesis by inducing cell cycle arrest and apoptosis, and suppressing tumor metastasis through blocking the PI3K/Akt and MAPK pathways and promoting FOXO3a activity.

## MATERIALS AND METHODS

### Clinical samples and DNA isolation

With the approval of institutional review board and human ethics committee, a total of 178 paraffin-embedded PTC tissues were randomly obtained from the First Affiliated Hospital of Xi'an Jiaotong University. Twenty-three normal thyroid tissues were also obtained from this Hospital as control subjects. None of these patients received chemotherapy and radiotherapy before the surgery. Informed consent was obtained from each patient before the surgery. All of the tissues were histologically examined by two senior pathologists at Department of Pathology of the Hospital based on World Health Organization (WHO) criteria. Genomic DNA was isolated from paraffin-embedded tissues as previously described [33].

### Human thyroid cancer cell lines

Human thyroid cancer cell lines BCPAP, FTC133, IHH4, K1 and 8305C were from Dr. Haixia Guan (The First Affiliated Hospital of China Medical University, Shenyang, P.R. China). C643 was from Dr. Lei Ye (Ruijin Hospital, Shanghai, P.R. China). Human glioblastoma cell line U251 and melanoma cell line A375 were purchased from the Kunming Cell Bank of the Chinese Academy of Sciences (Kunming, P.R. China) and American Type Culture Collection (ATCC, Manassas, VA), respectively. Cells were all routinely cultured at 37°C in RPMI 1640 medium with 10% fetal bovine serum (FBS), except for FTC133 that was cultured in DMEM/Ham's F-12 medium (Invitrogen Technologies, Inc., CA). To test PAX3 inactivation by epigenetic mechanisms, cells were either treated with 5 μM DNA methyltransferase (DNMT) inhibitor 5-aza-2′-deoxycytidine (5-Aza-dC) for 5 days or 7.5 μM histone deacetylase (HDAC) inhibitor suberoylanilide hydroxamic acid (SAHA) for 24 h, during which period medium and agent were replenished every 24 h. The powder of 5-Aza-dC and SAHA were obtained from Sigma-Aldrich and Cayman Chemical, and dissolved in 50% acetic acid/50% PBS and dimethyl sulfoxide (DMSO), respectively. In addition, MEK inhibitor GSK1120212 was purchased from Selleck Chemicals, and was dissolved in DMSO for inhibition of Erk signal. The same volume of the vehicle was used as the control.

### RNA extraction, conventional RT-PCR and quantitative RT-PCR (qRT-PCR)

Total RNA was extracted from 17 pairs of frozen surgical PTC and matched non-cancerous thyroid tissues or cell lines using TRIzol reagent (Takara Inc., Dalian, P.R. China) according to the instructions of manufacturer. Total RNA (~500 ng) was converted to cDNA using PrimeScript RT reagent Kit (Takara Inc., Dalian, P.R. China) according to the instructions of the manufacturer. Conventional RT-PCR was carried out to amplify *PAX3* gene. *β-actin* was run in parallel as control. PCR products were resolved by 1.2% agarose gel electrophoresis and visualized by ethidium bromide staining. The qRT-PCR assay was performed as previously described [33]. The primer sequences were presented in Supplementary Table S1.

### Sodium bisulfite treatment and methylation-specific PCR (MSP)

The protocols of sodium bisulfite treatment and MSP were performed as described previously [24].

### Plasmid constructs and transfection

The *PAX3* expression plasmid (pcDNA3.1-PAX3) was obtained for Yingrun Biotechnology Inc., Changsha, P.R. China. Cells were transfected with pcDNA3.1-PAX3 or pcDNA3.1 (empty vector) using X-tremeGENE HP DNA Transfection Reagent(Roche) according to the instructions of the manufacturer.

### Western blotting

Cells were lysed in prechilled RIPA buffer containing protease inhibitors. Equal amounts of protein lysates were separated by SDS-PAGE and transferred onto PVDF membranes (Roche Diagnostics, Mannheim, Germany). The membranes were then incubated with specific primary antibodies at 4°C overnight. Anti-PAX3, anti-total-Erk1/2 (t-Erk), anti-Smac, anti-N-cadherin, anti-SUZ12 and anti-Histone H3 were purchased from Abcam, Inc. Anti-phospho-Akt^Ser473^ (p-Akt), anti-total Akt (t-Akt) and anti-phospho-Erk1/2 (p-Erk) were purchased from Bioworld Technology, co, Ltd. Anti-p53, anti-caspase-3 and anti-caspase-9 were purchased from Santa Cruz Biotechnology, Inc. Anti-E-cadherin, anti-Vimentin, anti-phospho-Rb^Ser811^ (p-Rb), anti-p16, anti-Rb, anti-ZIC1 and anti-p21 were purchased from Epitomics, Inc. Anti-Bak and anti-Bax were purchased from Abgent, Inc. Anti-GAPDH was purchased from Abmart, Inc. Anti-FOXO3a, anti-phospho-FOXO3a^Thr32^ (p-FOXO3a), anti-EZH2 and anti-tri-Methyl-Histone H3-Lys27 (H3K27me3) were purchased from Cell Signaling Technology, Inc. Anti-p27 was purchased from WanLeibio, Inc. This was followed by incubation with species-specific HRP-conjugated secondary antibodies from ZSGB-BIO, Inc. or CWbio, Inc., and immunoblotting signals were visualized using the Western Bright ECL detection system (Advansta, CA). The integrated density was measured using ImageJ image software (ImageJ version 1.44p, NIH, MD). Relative density of target bands was calculated and adjusted according to integrated density of GAPDH (or Histone H3), which was used as loading control.

### Cell proliferation and colony formation assay

The protocols of cell proliferation and colony formation were similarly performed as described previously [33].

### Cell cycle and apoptosis assays

The protocols for assessing cell cycle and apoptosis were similarly performed as described previously [33].

### *In vivo* tumorigenicity

Four- to five-week-old female athymic nude mice were purchased from SLAC laboratory Animal Co., Ltd., Shanghai, P.R. China. The mice were randomly divided into two groups (five mice per group). Tumor xenografts were established by subcutaneous inoculation of 3 × 10^6^ FTC133 cells transfected with the indicated plasmids into the right armpit region of nude mice. From day 3 post-injection, tumor size was measured every 2 days. Tumor volumes were calculated by the formula (length × width^2^ × 0.5). The mice were sacrificed after 15 days, and tumors were harvested and weighted. In addition, tumors obtained from representative animals were embedded in paraffin, sectioned at 5 μm, and stained with hematoxylin and eosin (H&E). Ki-67 (BD Biosciences) staining was performed to evaluate cell proliferation. All experimental procedures were approved by the Animal Ethics Committee of Xi'an Jiaotong University.

### Cell migration and invasion assays

The protocols for evaluating cell migration and invasion were similarly performed as described previously [33].

### Immunofluorescence staining

The indicated cells were cultured onto coverslips in 6-well plates until 70% confluence. Cells were then fixed with 4.0% paraformaldehyde in phosphate-buffered saline for 15 min, and blocked with 5% goat serum for 30 min. Next, the coverslips were incubated at 4°C with the indicated primary antibodies overnight. Anti-FOXO3a antibody was purchased from Cell Signaling Technology, Inc. Anti-E-cadherin and anti-Vimentin antibodies were purchased from Epitomics, Inc. Anti-N-cadherin antibody was purchased from Abcam, Inc. Subsequently, the coverslips were incubated with FITC-conjugated goat anti-rabbit secondary antibody (Abcam) or Cy3-conjugated goat anti-rabbit secondary antibody (Bioss, Beijing, P.R. China), dyed with Hoechst33342, and fixed in glycerol. The images were obtained with an Olympus IX71 microscope (Olympus, Tokyo, Japan), and color mergence was performed using ImageJ image software (ImageJ version 1.44p, NIH, MD).

### Statistical analysis

Statistical significance of differences between the results was assessed using a standard 2-tailed *t* test and Mann–Whitney *U* test, conducted using SPSS statistical package (16.0, Chicago, IL). The data are expressed as mean ± standard deviation (SD) of the mean as indicated. *P* < 0.05 was considered statistically significant.

## SUPPLEMENTARY MATERIALS


