# Supplementary material for: PAX3 is a novel tumor suppressor by regulating the activities of major signaling pathways and transcription factor FOXO3a in thyroid cancer
Source: Oncotarget. 2016 Jul 21;7(34):54744–57. doi: 10.18632/oncotarget.10753 (PMC5342378; doi:10.18632/oncotarget.10753)
Supplement: Supplementary file 1 [file oncotarget-07-54744-s001.pdf]

PAX3 is a novel tumor suppressor by regulating the activities of major signaling pathways and transcription factor FOXO3a in thyroid cancer

Supplementary Materials

Supplementary Table S1: Sequences of primers used in this study

Genebank (Ref. ID)	Genes	Forward primer (5′–3′)	Reverse primer (5′–3′)	Product length (bp)
**RT-PCR or qRT-PCR**				
NM_001291428.1	*Bax*	TTTCTGACGGCAACTTCAACTG	AGGAAGTCCAATGTCCAGCC	133
NM_138621.4	*Bim*	AGGACTTGGGGTTTGTGTTG	TCTGAGTGTGACCGAGAAGGT	330
NM_053056.2	*Cyclin D1*	GACCTTCGTTGCCCTCTGT	TGAGGCGGTAGTAGGACAGG	140
NM_001130823.2	*DNMT1*	GACCATCAGGCATTCTACCA	GCTTTACATTTCCCACACTCAG	201
NM_175629.2	*DNMT3a*	GACCCTCCAAAGGTTTACCC	CCCGACGTACATGATCTTCC	204
NM_006892.3	*DNMT3b*	AATGTGAATCCAGCCAGGAAA	ACTGGATTACACTCCAGGAACCG	191
NM_004360.4	*E-cadherin*	CAGGTCTCCTCTTGGCTCTG	GACCGGTGCAATCTTCAAAA	142
NM_000043.4	*Fas*	CATCTGGACCCTCCTACCTC	ACAGTCTTCCTCAATTCCAATCC	114
NM_001455.3	*FOXO3a*	ACAAACGGCTCACTCTGTCC	CAGTTCCCTCATTCTGGACCC	162
NM_001924.3	*GADD45a*	ATCCACTTCACCCTGATCCA	TTGATCCATGTAGCGACTTTCC	249
NM_004530.5	*MMP-2*	TTGCTGGAGACAAATTCTGG	AAGAAGTAGCTGTGACCGCC	148
NM_004994.2	*MMP-9*	ACGACGTCTTCCAGTACCGA	GCACTGCAGGATGTCATAGG	128
NM_004995.3	*MMP-14*	GTTGTCTCCTGCTCCCCCT	AGCCATATTGCTGTAGCCAG	105
NM_001792.4	*N-cadherin*	GTGCATGAAGGACAGCCTCT	GGCATACACCATGCCATCTT	123
NM_181457.3	*PAX3*	GCCGCATCCTGAGAAGTAAA	CTTCATCTGATTGGGGTGCT	147
NM_005611.3	*p130*	ACTTCAGCAACAGTCCTTCAAA	GCAAATTCTTTTTGCAGGTGA	128
NM_000077.4	*p16*	CGAATAGTTACGGTCGG	GGGTCGGGTGAGAGTGG	122
NM_000389.4	*p21*	ACCTGTCACTGTCTTGTACC	GTAGAAATCTGTCATGCTGGTC	119
NM_004064.4	*p27*	CTCTGAGGACACGCATTTGG	TTTCTTCTGTTCTGTTGGCTC	146
NM_000546.5	*p53*	GAGCGTGCTTTCCACGAC	TGTTTCCTGACTCAGAGGGG	123
NM_000321.2	*Rb*	CAGAAGGCAACTTGACAAGAGA	CCTTCTCGGTCCTTTGATTG	130
NM_003068.4	*Slug*	CTACAGCGAACTGGACACACA	GCCCCAAAGATGAGGAGTATC	200
NM_005985.3	*Snail*	TCCAGAGTTTACCTTCCAGCA	CTTTCCCACTGTCCTCATCTG	218
NM_003810.3	*TRAIL*	GTGTACTTTACCAACGAGCTG	TCTTTCTAACGAGCTGACGG	166
NM_000474.3	*Twist*	GTCCGCAGTCTTACGAGGAG	GTCTGAATCTTGCTCAGCTTGTC	148
NM_003380.3	*Vimentin*	CGAAAACACCCTGCAATCTT	CTGGATTTCCTCTTCGTGGA	133
NM_001101.3	*β-actin*	GCACAGAGCCTCGCCTT	GTTGTCGACGACGAGCG	93
NR_003286.2	*18S*	CGCCGCTAGAGGTGAAATTC	CTTTCGCTCTGGTCCGTCTT	52
**MSP**				
NG_011632.1	PAX3-M	TTAATTAGCGCGTGTTTTC	CCGAACTCGAAAACATTTAT	112
NG_011632.1	PAX3-U	TGTTTAATTAGTGTGTGTTTTT	CCAAACTCAAAAACATTTATTAA	112

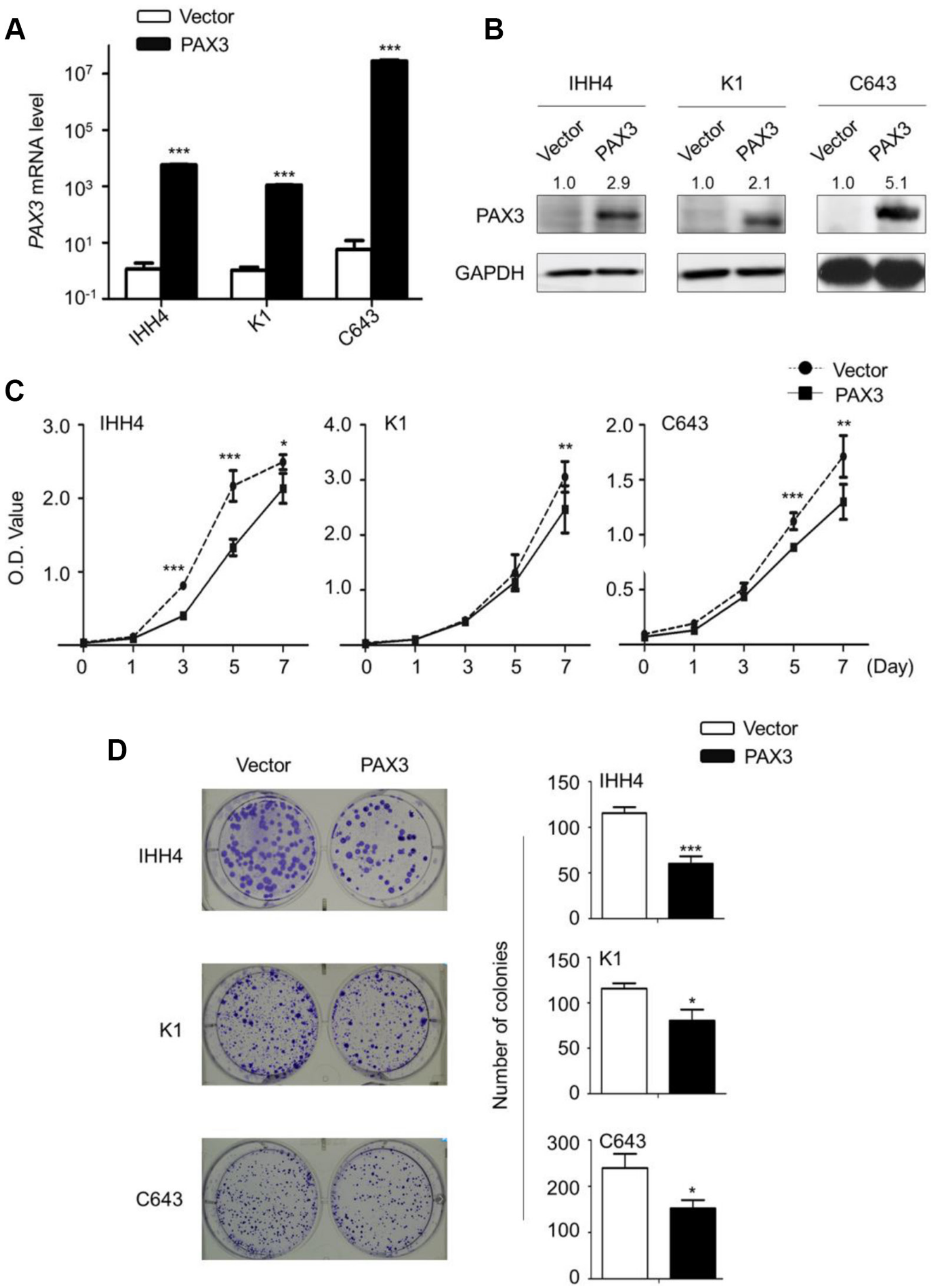

Supplementary Figure S1: PAX3 inhibits cell proliferation and colony formation in thyroid cancer cells. Ectopic expression of *PAX3* mRNA (**A**) and protein (**B**) in thyroid cancer cell lines IHH4, K1 and C643 was evidenced by qRT-PCR and western blot assays, respectively. *18S* rRNA was used as a normalized control for qRT-PCR assay. GAPDH was used as loading control in western blot assay. (**C**) PAX3 re-expression significantly inhibited thyroid cancer cell proliferation. (**D**) PAX3 re-expression inhibited colony formation of thyroid cancer cells. Left panel shows the representative images of colony formation in the indicated cells. Quantitative analysis of colony numbers is shown in the right panel. Data were presented as mean ± SD. Statistically significant differences were indicated: *$P < 0.05$; **$P < 0.01$; ***$P < 0.001$.

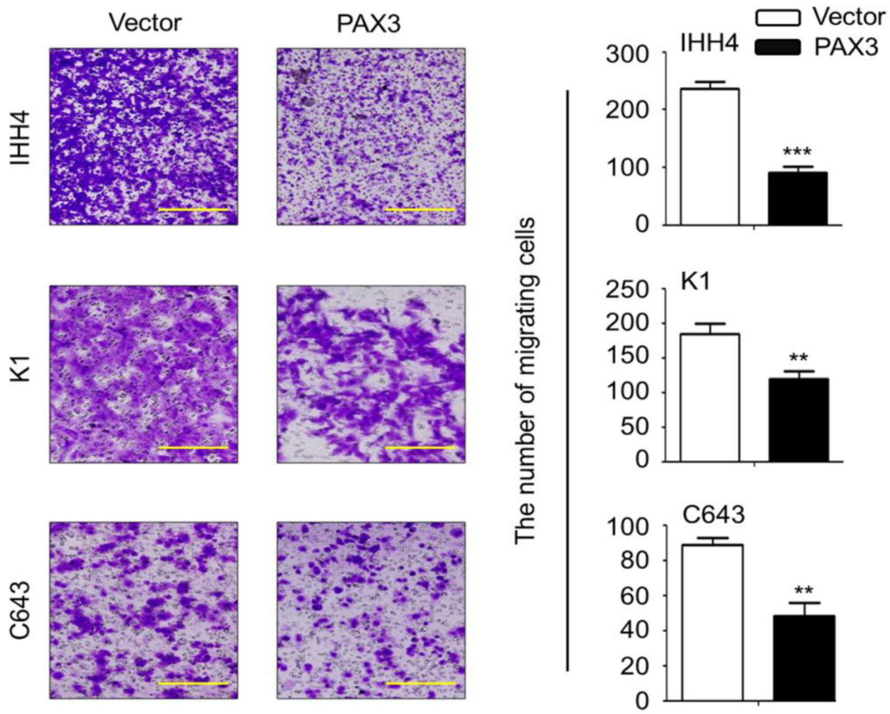

Supplementary Figure S2: PAX3 inhibits thyroid cancer cell migration. Ectopic expression of PAX3 inhibited cell migration in IHH4, K1 and C643 cells. The representative images of migrated cells (left panels). Histograms, corresponding to left panels, show means ± SD of cell numbers from three independent assays (right panels). Statistically significant differences were indicated: $**P < 0.01$; $***P < 0.001$.

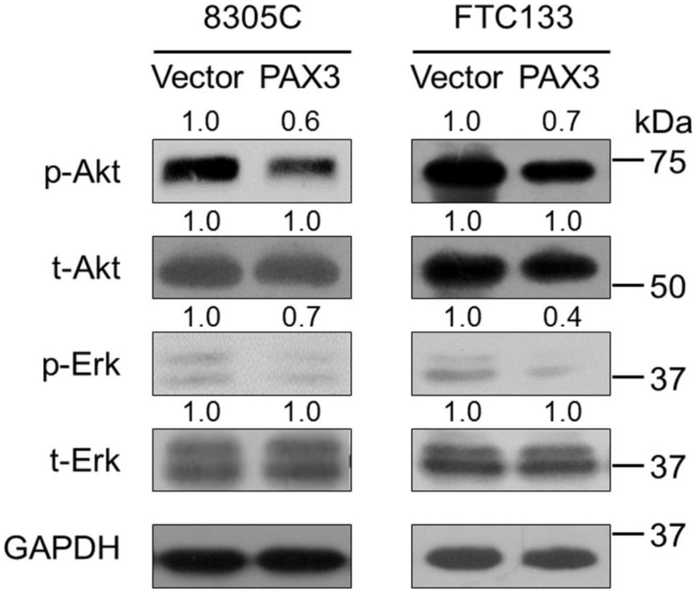

Supplementary Figure S3: PAX3 inhibits the activity of PI3K/Akt and MAPK/Erk pathways in thyroid cancer cells. The indicated cells were lysed. Supernatants were then collected and subjected to western blot analysis. The antibodies against phospho-Akt (p-Akt), total Akt (t-Akt), phospho-Erk (p-Erk) and total Erk (t-Erk) were used to test the effect of PAX3 re-expression on the activity of PI3K/Akt and MAPK/Erk pathways. GAPDH was used as loading control.

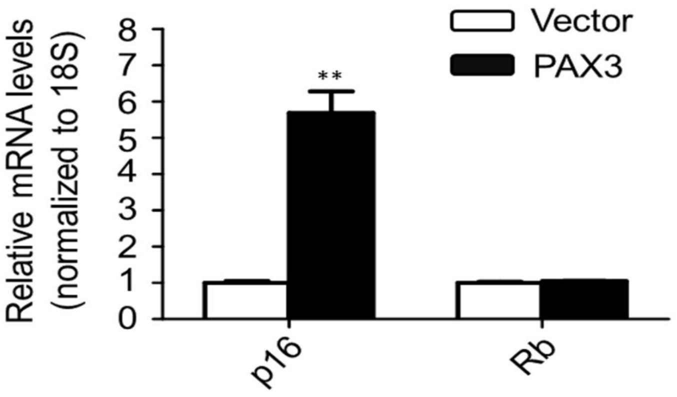

Supplementary Figure S4: qRT-PCR assay was performed to test the effect of ectopic expression of *PAX3* on mRNA expression of *p16* and *Rb* in the indicated cells. Their expression was normalized with *18S* rRNA levels. Data were presented as mean ± SD. Statistically significant differences were indicated: ***P* < 0.01.

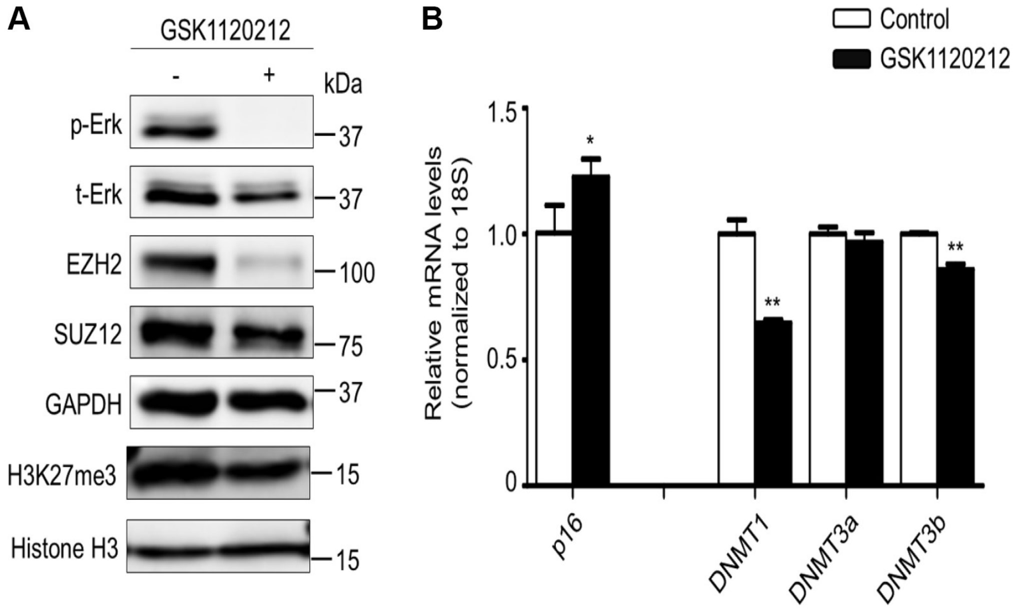

Supplementary Figure S5: MEK inhibitor up-regulates *p16* expression through epigenetic mechanisms via inhibiting the expression of DNMTs and PRC2 components EZH2 and SUZ12. (**A**) Western blot analysis was performed to evaluate the effect of MEK inhibitor GSK1120212 on H3K27me3 level and the expression of PRC2 components EZH2 and SUZ12 in BCPAP cells. GAPDH and Histone H3 were used as loading control. (**B**) qRT-PCR assay was used to test the effect of MEK inhibitor GSK1120212 on the expression of *p16*, *DNMT1*, *DNMT3a* and *DNMT3b* in BCPAP cells. Data were presented as mean ± SD. Statistically significant differences were indicated: **P* < 0.05; ***P* < 0.01.

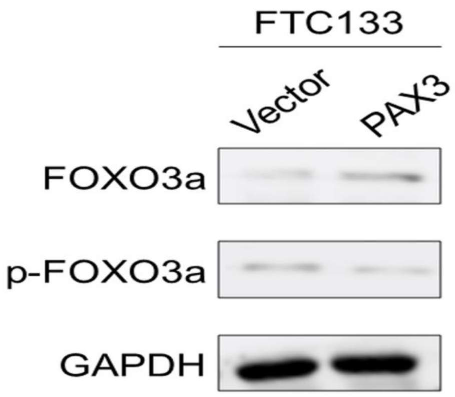

Supplementary Figure S6: Western blotting was used to test the effect of ectopic expression of PAX3 on the levels of FOXO3a protein and phosphorylated FOXO3a (p-FOXO3a) in FTC133 cells. GAPDH was used as loading control.

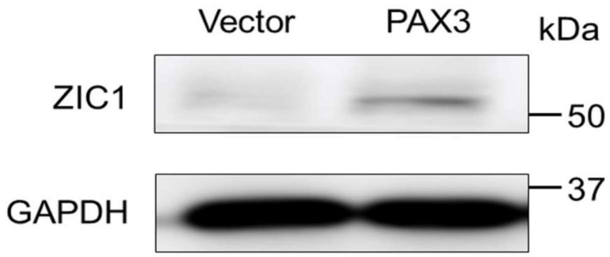

Supplementary Figure S7: Western blotting was performed to test the effect of ectopic expression of PAX3 on ZIC1 expression in BCPAP cells. GAPDH was used as loading control.

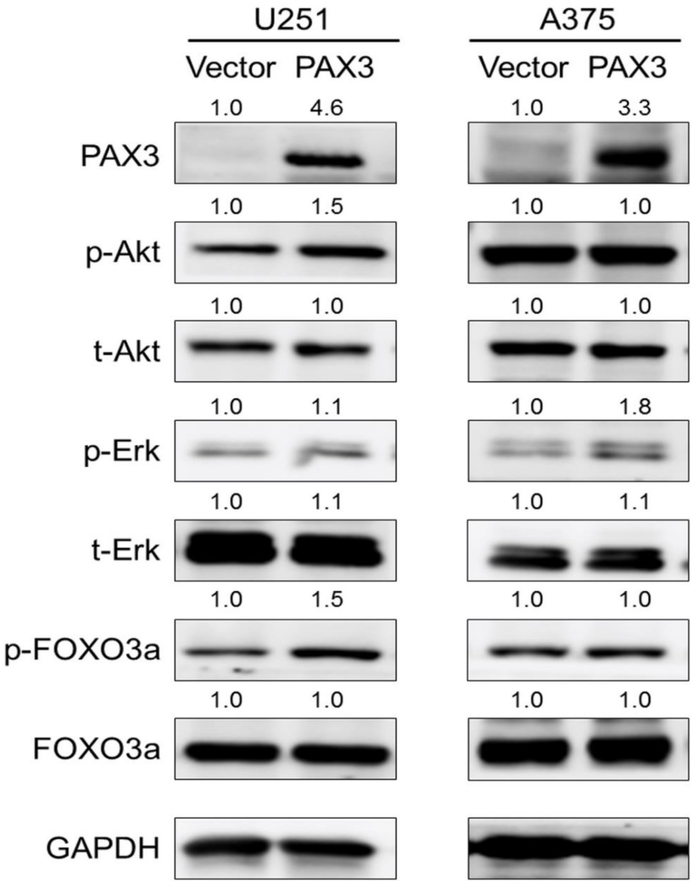

Supplementary Figure S8: Western blotting was used to test the effect of ectopic expression of PAX3 on the activities of PI3K/Akt and MAPK/Erk pathways and FOXO3a in glioblastoma cell line U251 and melanoma cell line A375. GAPDH was used as loading control.

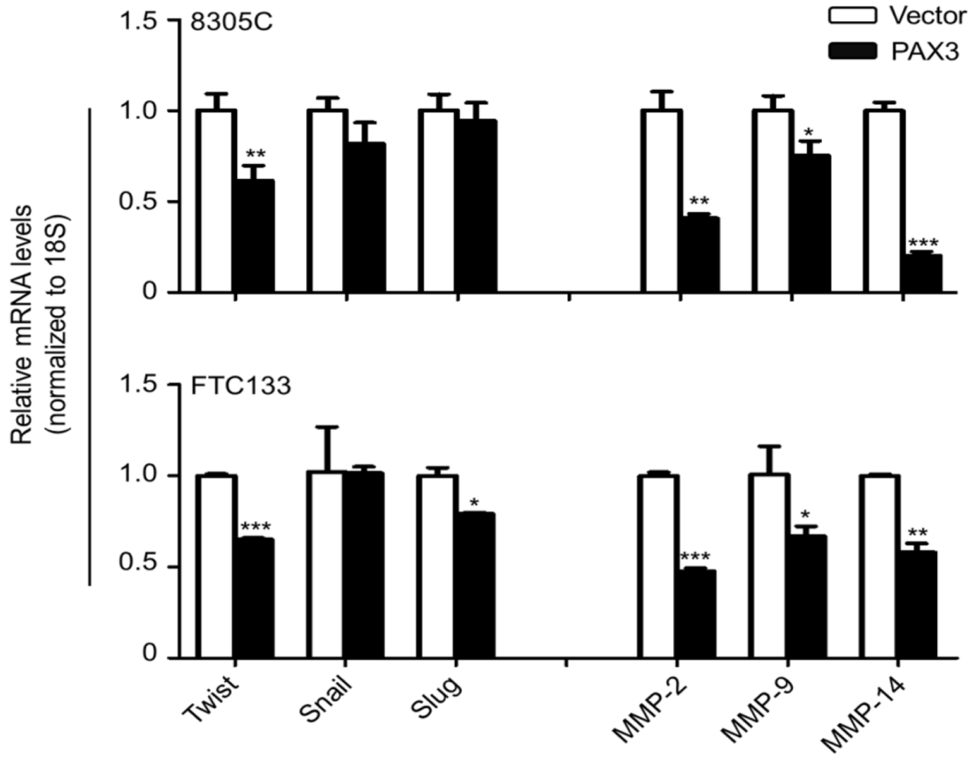

Supplementary Figure S9: qRT-PCR assay was performed to evaluate the effect of ectopic expression of PAX3 on the expression of E-cadherin transcription suppressors *Twist, Snail* and *Slug*, and metastasis-related genes *MMP-2, –9* and *–14* in 8305C and FTC133 cells. *18S* rRNA was used as a normalized control. Data were presented as mean ± SD. Statistically significant differences were indicated: **P* < 0.05; ***P* < 0.01; ****P* < 0.001.
